# PI(3,4,5)P3-mediated Cdc42 activation regulates macrophage podosome assembly

**DOI:** 10.1007/s00018-025-05664-2

**Published:** 2025-03-24

**Authors:** Yaoyue Qi, Cheng-han Yu

**Affiliations:** School of Biomedical Sciences, Li Ka Shing Faculty of Medicine, The University of Hong Kong, Pokfulam, Hong Kong

**Keywords:** Phosphoinositide, WASP, VAV1, Macrophage migration

## Abstract

Podosomes are adhesion structures with densely-polymerized F-actin. While PI(3,4,5)P3 and Cdc42-GTP are known factors to trigger WASP-mediated actin polymerization at the macrophage podosome, their causal mechanism to activate WASP remains unclear. Here, we demonstrate that spatially elevated Cdc42-GTP is a downstream effector of local PI(3,4,5)P3 production at the podosome. We further examine the expression and distribution of 19 Cdc42 guanine exchange factors (GEFs) and identify VAV1 as the key PI(3,4,5)P3-dependent Cdc42 GEF. VAV1 is spatially enriched at the macrophage podosome, and the association of VAV1 with the membrane plays a critical role in upregulating its GEF activity. Reintroduction of wildtype VAV1, rather than the PI(3,4,5)P3-binding deficient or catalytically dead mutants restores the matrix degradation and chemotactic migration of VAV1-knockdown macrophage. Thus, the biogenesis of PI(3,4,5)P3 acts as an upstream signal to locally recruit VAV1 and in turn triggers the guanine nucleotide exchange of Cdc42. Elevated levels of Cdc42-GTP then promote WASP-mediated podosome assembly and macrophage chemotaxis.

**Supplementary Information:**

The online version contains supplementary material available at 10.1007/s00018-025-05664-2.

## Introduction

The macrophage is a monocyte-derived cell type and often serves as the frontline of immune surveillance against pathogen infections in the connective tissue. To efficiently migrate through layers of extracellular matrix, macrophages assemble a distinct form of integrin-mediated adhesion known as podosome. Each podosome has a footprint of about 1 to 3 μm in diameter at the cell–matrix interface and comprises a central core that is abundant in branched actin filaments (F-actin) and a peripheral ring of adhesion-related molecules [1, 2]. Negatively charged phosphoinositides, such as PI(3,4,5)P3, are also locally enriched at the podosome [3]. In each macrophage, multiple podosomes are often assembled concurrently with a lifespan of a few minutes [4]. The periodic emergence of the podosome is indicated by the presence of a dot-like pattern of densely polymerized F-actin on the plasma membrane.

The intensive F-actin polymerization at the podosome core is collectively modulated by various actin regulators. In particular, WASP is one of the predominant Wiskott-Aldrich Syndrome family proteins expressed in hematopoietic cells and serves as the key nucleation promoting factor (NPF) to promote Arp2/3 dependent branched actin polymerization [5–7]. Arp2/3 structurally resembles monomeric actin and initiates the formation of a new branch of actin filament at a characteristic angle of 70° from the original one [8]. Inactive WASP is kept in an autoinhibited conformation in which the C-terminal VCA domain that triggers Arp2/3-mediated actin polymerization is blocked by the basic (B) region and GTPase-binding domain (GBD). When the B region and GBD interact with local phosphoinositide and GTP-bound Cdc42/Rac1 respectively, the VCA domain becomes accessible, and WASP is released from autoinhibited conformation and becomes activated [9, 10]. While neural WASP (N-WASP) and other WASP homologs exhibit similar functions as nucleation promoting factors, WASP plays an irreplaceable role in the podosome assembly and chemotactic migration of macrophage [5, 11].

Rho GTPases are membrane-associated small monomeric G proteins and dynamically regulate the assembly of cell adhesion and cytoskeleton. There are more than 20 members of Rho GTPases in mammalian cells [12]. Among them, Cdc42 acts as the master switch of filopodia formation, intracellular vesicle trafficking, and podosome assembly [5]. Like other G proteins, the activity of Cdc42 is dynamically regulated by guanine nucleotide-exchange factor (GEF) and GTPase activating protein (GAP). GEFs increase Cdc42 activity by promoting the exchange of bound GDP with GTP. GTP-bound Cdc42 can then activate downstream effectors, such as WASP and PAK family kinase [13, 14]. Conversely, GAPs decrease Cdc42 activity by accelerating GTP hydrolysis. GDP-bound Cdc42 fails to bind to the effector and therefore halts the downstream event. In general, GEFs exhibit specificity towards various GTPases in a context-dependent fashion. Membrane binding motifs within GEFs, such as the Pleckstrin homology (PH) domain, often govern their subcellular distributions and localized functions [15, 16]. Among more than 80 Rho GEFs identified, 37 GEFs are known to regulate Cdc42 activities [17, 18].

Dynamic organizations of membrane-bound signaling messengers, such as phosphoinositide and Cdc42 play a critical role in WASP-mediated F-actin polymerization [19, 20]. While the subcellular distribution of phosphatidylinositol kinase and Rho GEF can actively manipulate the local concentration of diffusive signaling messengers, the molecular mechanism and functional crosstalk between phosphoinositide production and GTPase regulation during the assembly of macrophage podosome remain unclear. In this study, we reveal the causation between the biogenesis of PI(3,4,5)P3 and Cdc42-GTP in monocyte-derived macrophage cells and the implication in the podosome assembly.

## Results

### WASP-Cdc42 interaction is essential for podosome assembly

**Fig. 1:**
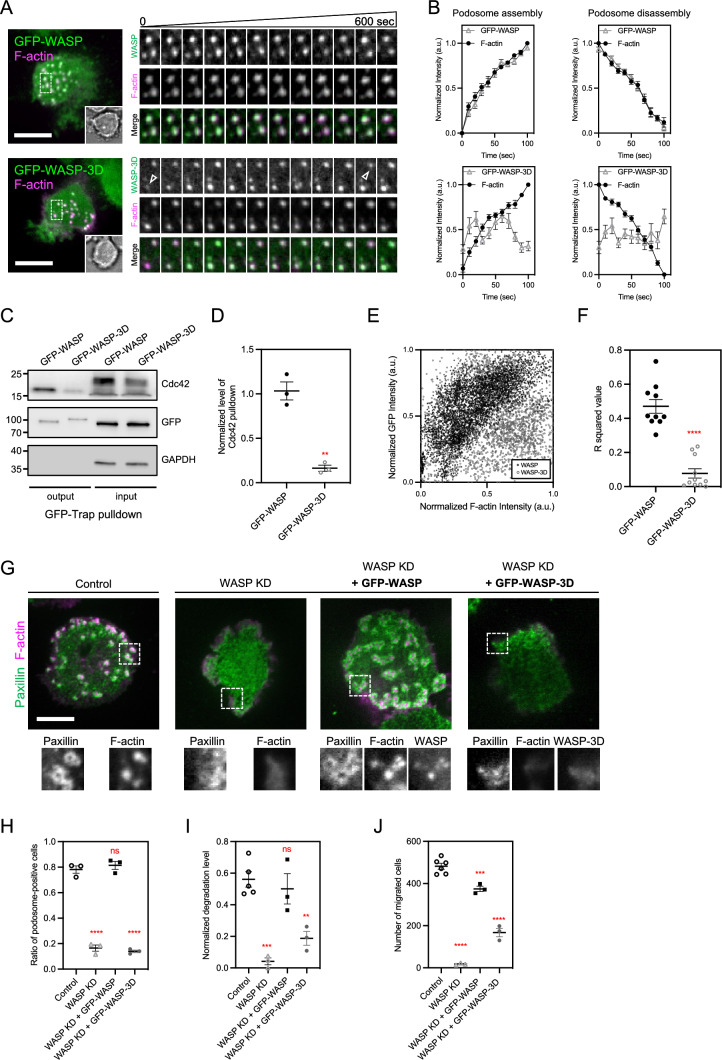
WASP-Cdc42 interaction is essential for podosome assembly. **A** GFP-WASP is steadily enriched at the podosome core labeled by F-actin marker lifeact-EBFP in THP-1 macrophage. While GFP-WASP-3D mutant also colocalizes with F-actin, its recruitment to podosome is unsteady and fluctuates (arrowheads). The insets represent the boxed regions. **B** The intensity levels of WASP, rather than WASP-3D closely follow the polymerization and depolymerization of F-actin at the podosome core (cell number N; N = 15 each). **C**, **D** The interaction between GFP-WASP-3D and Cdc42 is significantly reduced, compared to GFP-WASP. **E** Scatter plot of normalized intensities of F-actin and GFP-WASP or GFP-WASP-3D at each podosome. **F** R-squared value of linear regression of **E**. R-squared values of GFP-WASP-3D mutant are significantly lower than those of GFP-WASP (cell number N; GFP-WASP, N = 10; GFP-WASP-3D, N = 11). **G**, **H** Podosome assembly is suppressed in WASP-knockdown THP-1 macrophage, compared to the control. Podosome core and ring are identified by the staining of CF680R-phalloidin and anti-Paxillin, respectively. Reintroductions of wildtype GFP-WASP, rather than GFP-WASP-3D restore the podosome assembly. The insets represent the boxed regions. **I**, **J** Gelatin degradation and transwell migration are impeded in WASP-knockdown THP-1 macrophage, compared to the control. Expressing wildtype GFP-WASP, as opposed to GFP-WASP-3D reestablishes the gelatin degradation and transwell migration. See Figure S1. All experiments have been independently repeated three times. Error estimates are S.E.M. The statistical information is in Table S1. Unpaired two-tailed Student’s t-test and one-way analysis of variance (ANOVA) are used for the statistical analysis. not significant, ns; P > 0.1234; **P < 0.0021; ***P < 0.0002; and ****P < 0.0001. Scale bars represent 10 μm

Wildtype WASP and F-actin were visualized by TIRF microscopy and found enriched at the core of each podosome in THP-1 monocyte-derived macrophage (Fig. 1A, B). To investigate the functional role of Cdc42 in WASP-mediated podosome assembly, a Cdc42-binding deficient mutant, namely WASP-3D (F244D, H246D, H249D) was generated (Fig. 1C, D) [21]. While GFP-tagged WASP-3D can also be found at the podosome core, the level of GFP-WASP-3D, rather than wildtype GFP-WASP often fluctuated irrespective of F-actin and did not correlate with the event of podosome assembly and disassembly (Fig. 1A arrowhead and B). In addition, WASP-3D exhibited lower degrees of intensity correlation to F-actin, compared to wildtype WASP (Fig. 1E, F). Next, we sought to examine whether WASP-3D is sufficient to support podosome assembly in WASP-knockdown THP-1 macrophages. The assembly of each podosome can be identified by a dot-like F-actin core surrounded by a ring pattern of adhesion proteins, such as paxillin. In agreement with previous studies [5], WASP-knockdown macrophage showed very few podosomes (Figs. 1G, H and S1A, S1B). Gelatin degradation and serum-stimulated transwell migration of WASP-knockdown macrophages were also suppressed, in comparison with the control (Figs. 1I, J and S1C, S1D). Reintroduction of wildtype WASP, as opposed to WASP-3D, restored the assembly of podosome, gelatin degradation, and transwell migration. It appears that the association between WASP and Cdc42 is necessary to support the podosome assembly in the macrophage.

### PI(3,4,5)P3 production elevates Cdc42-GTP level at the podosome

**Fig. 2:**
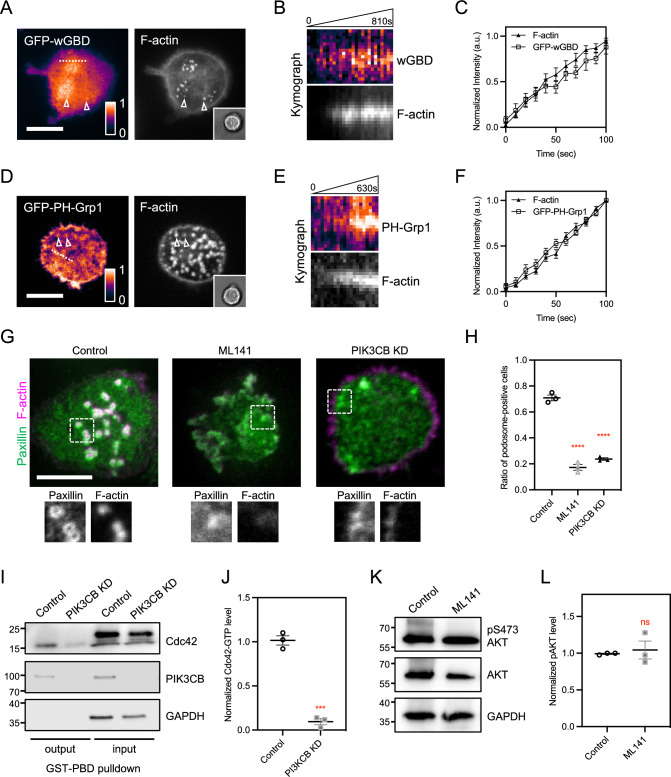
The production of PI(3,4,5)P3 acts as an upstream signal to promote Cdc42-GTP levels at the podosome. **A** GFP-wGBD is concentrated at the podosome in THP-1 macrophages (arrowheads). Dot-like F-actin assembly of podosome is marked by lifeact-EBFP. The inset represents the bright field image of the cell. **B** Kymographs of wGBD and F-actin along the dashed line in **A**. **C** The intensity levels of wGBD progressively increase as F-actin polymerizes at the podosome (cell number N; N = 10). **D** EGFP-PH-Grp1 is locally enriched at the podosome core labeled by lifeact-EBFP in THP-1 macrophage (arrowheads). The inset represents the bright field image of the cell. **E** Kymographs of PH-Grp1 and F-actin along the dashed line in **D**. **F** The intensity levels of PH-Grp1 steadily elevate as F-actin polymerizes at the podosome (cell number N; N = 10). **G**, **H** ML141 (20 µM) or shRNA knockdown of PIK3CB suppresses the podosome assembly, compared to the DMSO control. Podosome core and ring are identified by the staining of CF680R-phalloidin and anti-Paxillin, respectively. The insets represent the boxed regions. **I**, **J** Western blot analysis of Cdc42-GTP level via GST-PBD pulldown. Knockdown of PIK3CB results in the decrease of Cdc42-GTP level. **K**, **L** Western blot analysis of pS473-Akt level. ML141 does not block the S473 phosphorylation of Akt. All experiments have been independently repeated three times. Error estimates are S.E.M. The statistical information is in Table S1. Unpaired two-tailed Student’s t-test and one-way analysis of variance (ANOVA) are used for the statistical analysis. not significant, ns; P > 0.1234; ***P < 0.0002; and ****P < 0.0001. Scale bars represent 10 μm

Genetically encoded biosensors, such as wGBD can report the spatial distribution of GTP-bound Cdc42 [22]. When expressed, GFP-wGBD was specifically enriched at the polymerizing F-actin in the podosome core (Fig. 2A–C). Previously, we have reported that local production of PI(3,4,5)P3 by PIK3CB acted as the critical signal to promote podosome assembly in RAW 264.7 mouse macrophage [23]. Indeed, PH-Grp1, a specific PI(3,4,5)P3 probe, was also enriched at the polymerizing F-actin in the podosome core of THP-1 human macrophage (Fig. 2D–F). PIK3CB, the major isoform of PI3K in THP-1 macrophage, was also found at the podosome core (Fig. S2A–S2C). Next, we sought to investigate the causation between Cdc42-GTP and PI(3,4,5)P3 in promoting podosome assembly. ML141 is a selective allosteric inhibitor of Cdc42 (IC_50_ = 0.2 µM) and can also block Rac1 at a higher concentration (IC_50_ = 100 µM) [24]. When 20 µM of ML141 was applied to THP-1 cells, the level of Cdc42-GTP, rather than that of Rac1-GTP was suppressed (Fig. S2D–S2G). Cdc42-GTP inhibition (ML141, 20 µM), PIK3CB knockdown, or the expression of dominant-negative Cdc42-T17N mutant all effectively impeded the podosome assembly (Figs. 2G, H and S2H–S2J). The inhibition of Rac1-GTP by EHT1864 (5 µM), however, did not suppress the podosome assembly (Fig. S2K–S2N). Intriguingly, we found that the knockdown of PIK3CB, confirmed by the decrease of S473 phosphorylation of Akt (pS473-Akt) (Fig. S2O–S2P), resulted in the suppression of Cdc42-GTP level (Fig. 2I–J). On the other hand, ML141 treatment (20 µM) did not inhibit PI3K activity since the level of pS473-Akt remained unchanged (Fig. 2K, L). Therefore, the production of PI(3,4,5)P3 is an upstream signal to promote the level of Cdc42-GTP.

### VAV1 is a key PI(3,4,5)P3-dependent Cdc42 GEF in macrophage

**Fig. 3:**
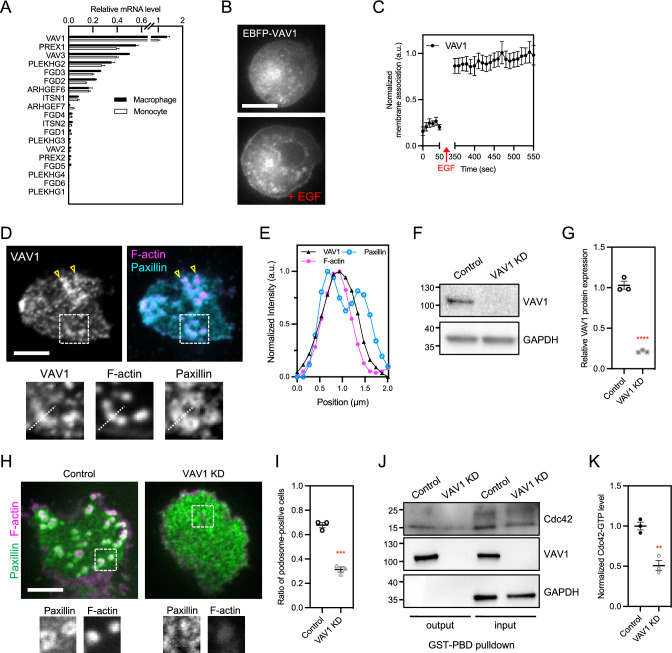
Identification of VAV1 as a PI(3,4,5)P3-dependent Cdc42 GEF. **A** RT-qPCR analysis of GEF expression in pre-differentiated monocyte and TGF-β1 induced macrophage. Among 19 Cdc42 GEFs, VAV1 exhibits the highest expression. **B**, **C** EBFP-VAV1 is actively recruited to the plasma membrane upon the stimulation of EGF (100 ng/mL) in MEF. **D** Endogenous VAV1 is enriched at the podosome in THP-1 macrophage. Podosome core and ring are identified by the staining of CF680R-phalloidin and anti-Paxillin, respectively. The insets represent the boxed regions. Anti-VAV1 staining can be found at the podosome core (inset), as well as other paxillin-positive adhesion sites (arrowheads). **E** Intensity line scan along the dashed line in **D**. **F**, **G** Western blot analysis to confirm the knockdown of VAV1. **H**, **I** Podosome assembly is suppressed in VAV1-knockdown THP-1 macrophage, compared to the control. The insets represent the boxed regions. **J**, **K** Knockdown of VAV1 results in the decrease of Cdc42-GTP level. All experiments have been independently repeated three times. Error estimates are S.E.M. The statistical information is in Table S1. Unpaired two-tailed Student’s t-test and one-way analysis of variance (ANOVA) are used for the statistical analysis. **P < 0.0021; ***P < 0.0002; and ****P < 0.0001. Scale bars represent 10 μm

As the level of Cdc42-GTP depends on the PI3K activity, we hypothesized that concentrated PI(3,4,5)P3 spatially recruited Cdc42 GEFs to promote the level of Cdc42-GTP at the macrophage podosome. In addition, macrophages natively formed podosomes, but monocytes, the precursor of macrophages were nonadherent and lacked podosomes. Thus, we sought to compare the expression profiles of Cdc42 GEF between pre-differentiated THP-1 monocyte and TGF-β1 induced THP-1 macrophage [25]. More than 35 GEFs have been reported to promote the guanine nucleotide exchange of Cdc42 [18, 26]. Among them, VAV, FGD, PREX, PLEKHG, and ITSN protein families contained phosphoinositide-binding PH domains, and ARHGEF6 and ARHGEF7 were localized at integrin-mediated adhesions. Therefore, we examined the expression profiles of 19 GEF candidates, including VAV1/2/3, FGD1/2/3/4/5/6, PREX1/2, PLEKHG1/2/3/4, ITSN1/2, and ARHGEF6/7 by reverse transcription quantitative real-time polymerase chain reaction (RT-qPCR). We found that the expression levels of VAV1, VAV3, PREX1, PLEKHG2, FGD3, and FGD2 were notably higher and increased after monocyte-to-macrophage differentiation (Fig. 3A).

Next, we adapted the approach of epidermal growth factor (EGF) stimulated PI3K activation to examine the PI(3,4,5)P3 dependent spatial redistribution of shortlisted GEFs. Specifically, we constructed and transfected the plasmids of fluorescent protein-fused VAV1, VAV3, PREX1, PLEKHG2, FGD3, and FGD2 into mouse embryonic fibroblasts (MEFs). We then used a spinning-disk confocal microscope to monitor the cross-section of MEF’s great circle and measured the dynamical recruitment of GFP-fused GEFs on the plasma membrane before and after the EGF stimulation [27]. We found that the levels of VAV1 and VAV3 at the plasma membrane promptly increased after the addition of EGF (100 ng/mL), while those of PREX1, PLEKHG2, FGD3, and FGD2 did not (Figs. 3B, C and S3A–S3L). Since VAV1 was homologous to VAV3 and exhibited a higher expression level, we sought to examine the functional role of VAV1 in the signal axis of PI(3,4,5)P3-Cdc42 mediated podosome assembly.

### Knockdown of VAV1 suppresses the assembly of macrophage podosome

In wildtype THP-1 macrophages, endogenous VAV1 mainly colocalized with F-actin at the podosome core and was surrounded by paxillin (Fig. 3D, E). Low levels of VAV1 can also be found at other adhesion sites and podosome ring. In alignment with the prior observation, the levels of VAV1 on the plasma membrane of both adhesion and non-adhesion area are significantly reduced in PIK3CB-depleted THP-1 macrophages, compared to the control (Fig. S4A). When endogenous VAV1 was knocked down, macrophages lost prominent dot-like F-actin assembly but were still able to adhere to the fibronectin-coated substrate (Fig. 3F–I). However, the number of podosome-positive cells and the level of Cdc42-GTP were significantly reduced in VAV1-knockdown THP-1 macrophages, compared to the control (Fig. 3H–K). It appears that VAV1 is an essential factor in promoting the assembly of macrophage podosome.

### Membrane association and GEF activity of VAV1 are both necessary to support podosome assembly and transwell migration

**Fig. 4:**
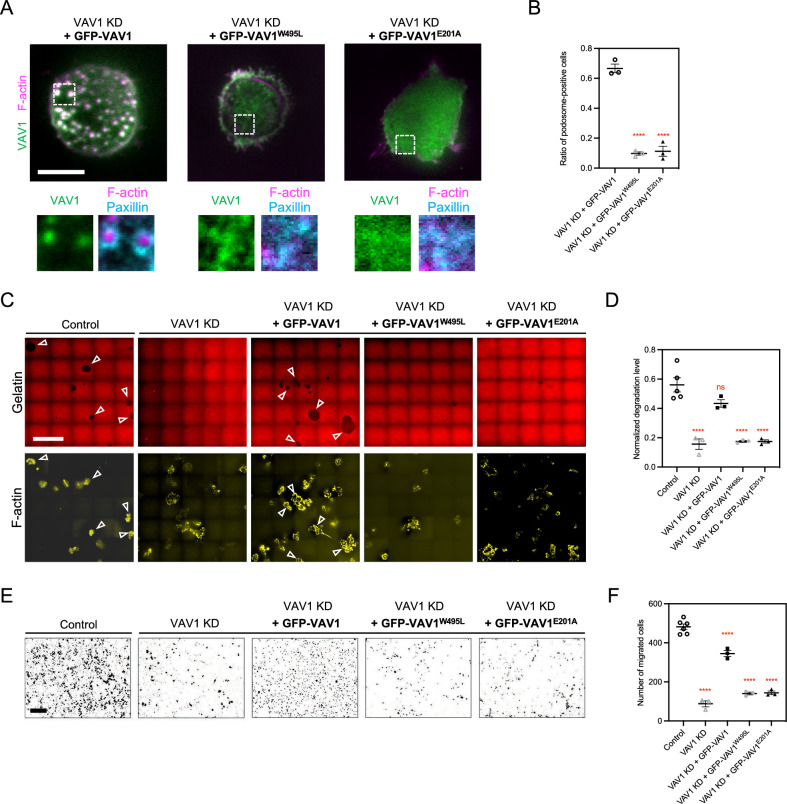
Membrane association of VAV1 is essential for macrophage migration and gelatin degradation. **A**, **B** Reintroductions of wildtype GFP-VAV1, rather than GFP-VAV1-W495L or GFP-VAV1-E201A restore the podosome assembly in VAV1-knockdown THP-1 macrophages. Podosome core and ring are identified by the staining of CF680R-phalloidin and anti-Paxillin, respectively. The insets represent the boxed regions. **C**, **D** Degradation of Cy3-labeled gelatin in the indicated conditions. Representative images are stitched from 5 × 5 tile scan. Gelatin degradation activity of VAV1-knockdown THP-1 macrophage is suppressed, compared to the control. Expressing wildtype GFP-VAV1, as opposed to VAV1-W495L or GFP-VAV1-E201A restores the gelatin degradation (arrowheads). F-actin is stained by CF680R-phalloidin. **E**, **F** Representative images of transwell-migrated THP-1 macrophages in the indicated conditions. Crystal violet is used to stain the cells. Transwell migration of VAV1-knockdown THP-1 macrophages is impeded, compared to the control. Reintroductions of wildtype GFP-VAV1, rather than VAV1-W495L or GFP-VAV1-E201A promote the transwell migration. All experiments have been independently repeated three times. Error estimates are S.E.M. The statistical information is in Table S1. One-way analysis of variance (ANOVA) is used for the statistical analysis. not significant, ns; P > 0.1234 and ****P < 0.0001. Scale bars represent 10 μm in A, 50 μm in **C**, and 200 μm in **E**

**Fig. 5:**
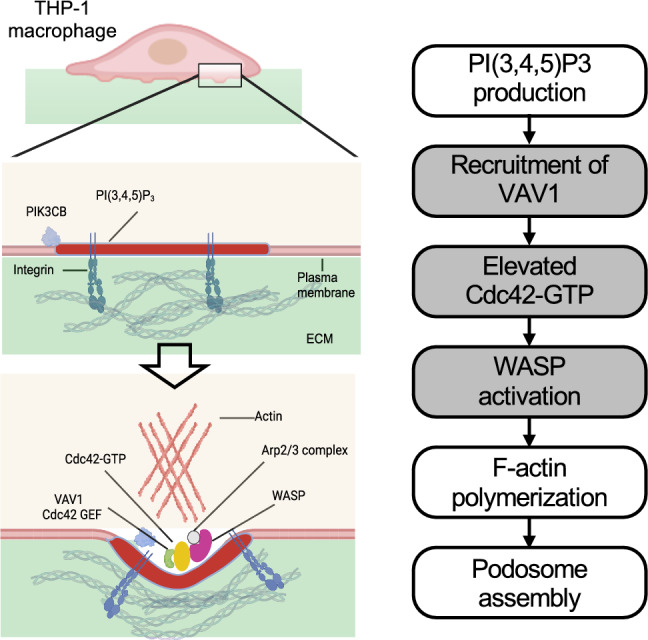
Summary of PI(3,4,5)P3-VAV1-Cdc42 signaling cascade. The production of PI(3,4,5)P3 by PIK3CB is an upstream signal to recruit VAV1 to the plasma membrane. Activated VAV1 then promotes the level of GTP-bound Cdc42. Together with anionic lipids, locally concentrated Cdc42-GTP activates WASP-mediated podosome assembly in the macrophage

To examine the importance of VAV1’s GEF activity in the podosome, the GEF-dead mutant GFP-VAV1-E201A was generated [28]. In addition, the membrane-binding deficient mutant GFP-VAV1-W495L was constructed [29], and its absence from the plasma membrane upon EGF stimulation was confirmed (Fig. S4B–S4C). Introduction of either VAV1-E201A or VAV1-W495L mutants into VAV1-knockdown THP-1 macrophage failed to reestablish the podosome assembly, compared to the wildtype VAV1 (Fig. 4A, B). Next, we examined the functional roles of VAV1 in gelatin degradation and serum-stimulated transwell migration of macrophage. VAV1-depleted THP-1 macrophages exhibited significant decreases in the area of degraded gelatin and the number of cells migrating through the Matrigel-coated membrane. Reintroduction of wildtype VAV1, rather than VAV1-E201A or VAV1-W495L mutants, effectively restored the area of gelatin degradation and transwell migration (Fig. 4C–F). Intriguingly, the phosphorylation level of Y160, a key indicator of GEF activity [30] was also reduced in VAV1-W495L membrane binding mutant, in comparison with wildtype VAV1 (Fig. S4D, S4E). Thus, membrane associated VAV1 with a functional GEF motif is vital for macrophages to perform the physiological activities of matrix degradation and chemotaxis (Fig. 5).

## Discussion

Phosphoinositides and GTPases are diffusive molecules on the plasma membrane and act as critical signals to initiate WASP-mediated actin polymerization at the podosome. Previously, we have reported that PI(3,4,5)P3 is one of the key phosphoinositides enriched at the podosome [3, 27, 31]. The crosstalk between phosphoinositide biogenesis and GTPase regulation has been widely speculated [32, 33]. Here, we reveal their causal relationship and demonstrate that the production of PI(3,4,5)P3 mediated by PIK3CB is an upstream signal to upregulate the level of Cdc42-GTP. In addition, we identify VAV1 as the key PI(3,4,5)P3-dependent Cdc42 GEF. We demonstrate that the spatiotemporal membrane association of VAV1 plays a critical role in promoting its GEF activity. Specifically, wildtype VAV1, as opposed to the membrane binding deficient VAV1-W495L mutant can restore podosome assembly, gelatin degradation, and chemotactic migration of VAV1-knockdown macrophages.

In THP-1 macrophages, VAV1 expression is significantly higher than VAV3, and the transcripts of VAV2 are almost absent. Among all 19 GEFs we examined, only VAV1 and VAV3 exhibit prompt recruitment to the plasma membrane upon EGF stimulation. As VAV1 and VAV3 share high degrees of domain similarity [34], it is believable that VAV3 may play a compensation role and support the podosome assembly in VAV1-knockdown cells. The activation mechanism of autoinhibited VAV1 has been reported [35]. Initially, the key helix from the acidic region (Ac) between calponin homology (CH) and Dbl homology (DH; responsible for GEF enzymatic activity) domains of VAV1 binds to the catalytic surface and blocks the substrate loading. The phosphorylation of Y142, Y160, and Y174 residues in the Ac domain by Syk or Src family kinases subsequently relieves the autoinhibition [36, 37]. High-resolution NMR structure data have mechanistically demonstrated the modulatory interactions among CH, DH, and PH domains of autoinhibited VAV1 and revealed the intramolecular steps to create kinase-accessible conformations to phosphorylate these key tyrosine residues in the Ac domain [38]. In addition, the D406A mutation in the PH domain of VAV1 has been shown to destabilize such modulatory interaction with the CH domain and led to an increase in GEF activity. The interaction between the PH domain of VAV1 and PI(3,4,5)P3 has been reported [39, 40]. However, the overall conformational change of VAV1 induced by PI(3,4,5)P3 binding has not been explicitly investigated [41]. Here, we find that the phosphorylation level of Y160 of membrane-binding deficient mutant VAV1-W495L is significantly reduced, in comparison to the wildtype VAV1. Therefore, membrane binding of VAV1 is crucial to positively support the phosphorylation of tyrosine residues in the Ac domain and promote the GEF activation.

The specificities of GEF and Rho GTPase are often in a context-dependent manner [18]. For example, VAV1 can promote the guanine nucleotide exchange of Cdc42 and Rac1 in HUVEC endothelial cells [42] and has been shown to play a redundant role with VAV3 during the platelet activation [43]. In dendritic cells, VAV1 also promotes the adherence to fibronectin and positively regulates the adhesion-dependent signaling [44]. In pancreatic cancer cells, VAV1 triggers the guanine nucleotide exchange of Cdc42 and is a major driver of invadopodium assembly [45]. Likewise, VAV2 reacts with Cdc42, Rac1, and RhoA in NIH3T3 fibroblasts [46] and promotes the level of Rac1-GTP in lung epithelial cells [47]. In podosome-forming cells, the level of RhoA-GTP is downregulated by RhoA GAP ARAP3(3) and an increase in RhoA-GTP level results in podosome disassembly [48]. Thus, the contribution of VAV1 in promoting the RhoA-GTP level seems to be inconsequential during the assembly of macrophage podosome (data not shown). On the other hand, VAV1 presumably can react to both Cdc42 and Rac1 in the macrophage, but the specific inhibition of Cdc42, rather than Rac1 can suppress the podosome assembly. This is probably due to the preferential binding of WASP to Cdc42, in comparison with Rac1 [49–51].

Other Cdc42 GEFs, including PREX1, PLEKHG2, and FGD family have previously been shown to bind to PI(3,4,5)P3 [52–54]. In addition, ARHGEF6 (alpha-PIX; hematopoietic specific) and ARHGEF7 (beta-PIX; ubiquitously expressed) are Cdc42 GEFs associated with integrin-mediated adhesions and also contain the PH domain [55]. However, we do not observe distinct enrichment of these GEFs to the plasma membrane upon EGF-stimulated PI3K activation. As the expression of VAV1 is significantly higher in THP-1 monocytes and increases further after the monocyte-to-macrophage differentiation, the contributions from other GEFs mentioned above to the PI(3,4,5)P3 and Cdc42 dependent podosome assembly are probably less critical.

Metastatic cancer cells often assemble the invadopodium that contains many molecular components identical to podosome. In particular, the activation of PI3K at the invadopodium has been shown to play a pivotal role in AKTIP and SNX9 dependent endocytosis of membrane receptors [31]. While the molecular determinants, such as phosphoinositide and GTPase effectors between macrophage podosome and cancer invadopodium are indeed similar, the functional outcome in invasive migration can be distinctly different. In general, a better understanding of the feedback loop involving phosphatidylinositol phosphatase, Rho GAP, and Rho GDI will be necessary to unravel the overarching mechanism of cytoskeletal reorganization and invasive cell migration.

## Materials and methods

### Cell culture

Human monocytic THP-1 cells were obtained from the American Type Culture Collection and cultured in RPMI 1640 media (Gibco), supplemented with 10% fetal bovine serum (FBS, heat inactivated) by volume and 2-mercaptoethanol (50 μM). THP-1 cells were differentiated using transforming growth factor beta 1 (TGF-β1, 100 nM; Sigma-Aldrich, T7039) for 24 h. RPTPα + / +mouse embryonic fibroblast (MEF) cells were kindly provided by Jan Sap from New York University. The MEF cells were cultured in DMEM, supplemented with 10% FBS by volume. The cells were incubated at 37 °C with 5% CO_2_. For the live cell imaging, a glass-bottom dish was precoated with fibronectin (10 μg/mL; Gibco, 33016015) for 30 min at 37 °C. Cells were then seeded on the glass-bottom dish and cultured in Leibovitz’s L-15 medium (Gibco, 21083027).

### RT-qPCR analysis

Total RNA was extracted from the cells using RNeasy Plus Mini Kit (Qiagen, 74,104). The purity and concentration of the extracted RNA were assessed using a NanoDrop spectrophotometer, and only samples meeting the quality criteria (A260/A280 ratio between 1.8 and 2.0) were used for further analysis. Next, 1 μg of the high-quality total RNA was reverse transcribed into complementary DNA (cDNA) using a reverse transcription kit (Takara, RR086A). Reverse transcribed cDNA was then used as the template for quantitative real-time PCR experiments to quantify the expression levels of the target genes. The RT-qPCR reactions were performed using gene-specific primers and TB Green Premix Ex Taq™ II (Takara, RR82WR). The reactions were carried out on a CFX96 real-time PCR system (Bio-Rad) with the following thermal cycling conditions: 2 min of initial denaturation at 95 °C, followed by 40 cycles of 15 s at 95 °C (denaturation) and 1 min at 60 °C (combined annealing and extension). The relative expression levels of the target genes were normalized to the expression of β-actin. The information of qPCR primers can be found in Table S4.

### Plasmid construction

Full-length human Wiskott-Aldrich syndrome protein (WASP) was a gift from Gareth Jones. EGFP-Grp1-PH was a gift from Guillaume Halet. Lifeact-EBFP2 was a gift from Pakorn Kanchanawong. EGFP-Cdc42 (plasmid #20142), pC.HA VAV1 (plasmid #14553), pC.HA VAV3 (plasmid #14555), GFP-wGBD (plasmid #26734) and pDONR223-PIK3CB (plasmid #23543) were obtained from Addgene. FGD2 (HsCD00040702), FGD3 (HsCD00296965), PLEKHG2 (HsCD00744615), PREX1 (HsCD00862343), and ARHGEF6 (HsCD00964078) entry clone plasmids were obtained from DNASU. Plasmids were subcloned into various expression vectors, including pDest-EGFP-N1, pDest-mCherry-N1, EGFP-C1, EBFP-C1, or pLenti-CMV-MCS-GFP-SV-Puro (Addgene #73572). Point mutants, including WASP-3D (F244D, H246D, H249D), Cdc42-T17N, VAV1-W495L, and VAV1-E201A were generated by PCR. Scramble and shRNA oligos were obtained from IDT and subcloned into pLKO.1-TRC (Addgene #10878) or pLKO-mCherry-luc-puro (Addgene #29784) lentiviral-based vectors. The information of PCR primers and shRNA sequences can be found in Tables S2 and S3.

### Transfection

Lentiviral-based approach was used to transfect THP-1 cells. The plasmid of interest, packaging plasmid (psPAX2; Addgene #12260), and envelope plasmid (pMD2.G; Addgene #12259) were diluted in serum-free medium and then mixed with the transfection reagent PolyJet (SignaGen Laboratories, SL100688) for 15 min. The transfection mixture was then dropwise added to 293 T cells. After 4–6 h, the medium was replaced with fresh complete growth medium. Virus-containing supernatant was harvested at 48- and 72-h post-transfection and then was passed through a 0.22 μm filter to remove cellular debris. The filtered virus-containing media, supplemented with polybrene (10 μg/mL; Sigma, TR-1003) to enhance transduction efficiency, was subsequently added to THP-1 cells. After 48 h, puromycin was introduced to select the transfected cells. Electroporation-based Neon transfection system (Life Technologies) was used to achieve transient transfection. Cells were resuspended in Opti-MEM reduced serum medium and mixed with the desired plasmid DNA. The cell-DNA suspension was then electroporated with the condition of 1200 V and 20 ms pulse. After electroporation, cells were transferred to pre-warmed complete growth medium and cultured for 24–48 h before the subsequent experiment.

### Antibodies and reagents

The primary antibodies included anti-WASP (Cell Signaling Technology 4860; 1:1000), anti-GFP (Cell Signaling Technology 2956; 1:1000), anti-PIK3CB (Proteintech 67121-1-Ig; 1:2000), anti-AKT (Cell Signaling Technology 4691; 1:1000), anti-Phospho-Akt (Ser473) (Cell Signaling Technology 4060; 1:1000), anti-VAV1 (Invitrogen MA5-31488; 1:1000 for western blotting, 1:100 for immunofluorescence), anti-Rac1 (BD Biosciences 610651; 1:1000), anti-Cdc42 (Proteintech 10155-1-AP; 1:1000) and anti-GAPDH (Thermo Fisher Scientific AM4300; 1:10000). The secondary antibodies used were anti-mouse HRP (Cell Signaling Technology 7076; 1:2000) and anti-rabbit HRP (Cell Signaling Technology 7074; 1:2000), AF594-anti-mouse (Thermo Fisher Scientific A-21203; 1:500), AF594-anti-rabbit (Thermo Fisher Scientific A-21207; 1:500), AF488-anti-mouse (Thermo Fisher Scientific A-21202; 1:500), and AF488-anti-rabbit (Thermo Fisher Scientific A-21206; 1:500). Actin filaments were stained by CF680R-phalloidin (Biotium 00048; 1:1000). ML141 and EHT1864 were purchased from Selleckchem (S7686 and S7482). Human EGF recombinant protein was purchased from Thermo Fisher Scientific (PHG0311).

### Western blot analysis

Cells were lysed using a cold RIPA buffer (Thermo Fisher Scientific, 89900) supplemented with protease and phosphatase inhibitors (Thermo Fisher Scientific, A32955). The protein concentration in the cell lysates was determined using a Bradford assay (Bio-Rad, 5000006). Equal amounts of protein (20–30 μg) were separated by SDS-PAGE on 10% gels (Bio-Rad, 1610173) and transferred onto PVDF membranes (Millipore, IPVH00005). The membranes were blocked with 5% non-fat milk in Tris-buffered saline with Tween-20 (TBST) for 1 h at room temperature. They were then incubated overnight at 4 °C with the primary antibody diluted in 5% BSA in TBST. After extensive washing with TBST, the membranes were incubated with HRP-conjugated secondary antibody for 1 h at room temperature. The blots were incubated with an enhanced chemiluminescence (ECL) substrate (Thermo Fisher Scientific, 345801), and the chemiluminescence signals are detected by ChemiDoc MP Imaging System (Bio-Rad). ImageJ software was used to quantify the band intensity. The protein levels were normalized to the expression of housekeeping proteins, to account for any variations in sample loading. The uncropped blots are listed in Figure S5.

### Cdc42 activity assay

Cells were first lysed using the lysis buffer provided in the Cdc42 activation assay kit (Biochem, BK034). The lysates were then incubated with beads conjugated with Cdc42-binding domain of PAK1 for 1 h at 4 °C. After the incubation, the beads were washed three times with the provided wash buffer to remove any unbound proteins. The bound Cdc42, as well as the total Cdc42 in the original cell lysate were detected by Western blotting using the primary antibody of Cdc42.

### Immunofluorescence

Freshly prepared paraformaldehyde (PFA, 4%) was used to fix the cells for 15 min at room temperature. The fixed cells were then permeabilized with Triton X-100 (0.05%) in phosphate-buffered saline (PBS, 1x) for 15 min and blocked with bovine serum albumin (BSA, 5%) in PBS for 1 h at room temperature. The cells were incubated with primary antibodies diluted in the blocking buffer overnight at 4 °C. After washing with PBS (3 times, 1 mL each), the cells were incubated with the fluorescently labeled secondary antibodies for 2 h. Unbound secondary antibodies were subsequently rinsed out using PBS (3 times, 1 mL each).

### Fluorescence microscopy

An iLas2-based TIRF microscope system equipped with an EMCCD camera (Photometrics Evolve 512) and 100 × oil immersion lens (NA 1.46) was used to visualize the macrophage podosome. The TIRF illumination was provided by AOTF-controlled solid-state lasers and controlled by MetaMorph software (Molecular Devices). An Inverted spinning-disk confocal microscope equipped with Yokogawa CSU-X1, an EMCCD camera (Hamamatsu C9100-23B) and 100 × oil immersion lens (NA 1.45) was used to visualize the membrane association. A piezo-based Z stage and AOTF-controlled solid-state lasers were controlled by Volocity software (Perkin-Elmer). To achieve long-term time-lapse imaging, an environmental chamber was attached to the microscope and maintained in the conditions of 37 °C and 5% CO_2_.

### Intensity analysis during the podosome assembly

Live THP-1 cells with fluorescent markers were visualized by a TIRF microscope. Time-lapsed images were acquired with a 10-s interval. In each podosome, a dot-like F-actin assembly was used to define the region of interest (ROI), and the intensities of the other molecule of interest within the ROI were measured using ImageJ or Imaris software. The charts of normalized intensity versus time were prepared by GraphPad software.

### Quantification of podosome-positive cells

The distinct core/ring organizational structures of podosomes were identified through fluorescence microscopy. The podosome core was labeled with F-actin markers, such as phalloidin or lifeact, while the podosome ring was labeled with the adhesion marker paxillin. Typically, the mean intensity value of F-actin staining at the podosome core was two times or more, compared to the cytosol background. A macrophage cell was defined as a podosome-positive cell if it contained more than ten podosomes. While other types of quantification, such as the number of podosomes per cell can also be utilized, intrinsic variations within each condition were often large and widely spread. Thus, the ratio of podosome-positive cells was used to evaluate the impact in various conditions.

### Quantification of membrane association

Fluorescent protein-fused Cdc42 GEFs were transiently transfected in MEFs. After 12 h serum starvation, the cells were trypsinized and resuspended in PBS with a membrane marker DiI (1,1′-Dioctadecyl-3,3,3′,3′-Tetramethylindocarbocyanine Perchlorate) for 20 min in a 37 °C incubator. The cells were then placed on an uncoated glass-bottom dish, and time-lapsed imaging of the GEF of interest and DiI was performed at a single confocal section of the great circle plane of the cell using a spinning-disk confocal microscope. EGF (100 ng/mL) was subsequently added to stimulate PI(3,4,5)P3 production. Using ImageJ software, the intensity of the Cdc42 GEFs on the plasma membrane (I_mem_) was measured by an outside-in line scan across the plasma membrane indicated by DiI. Camera background (I_bkg_) and cytosol background (I_cyt_) were measured accordingly. A self-defined parameter MA [membrane association; (I_mem_−I_cyt_)/(I_mem_−I_bkg_)] was used to quantify the membrane association of various Cdc42 GEFs. A higher MA value suggested a stronger association with the plasma membrane.

### Gelatin degradation assay

The glass-bottom dishes were incubated with poly-L-lysine (50 μg/mL) for 20 min and then washed three times with PBS. Next, the substrate was cross-linked with 0.25% (w/v) glutaraldehyde for 15 min, followed by three more PBS washes. The dishes were then incubated with Cy3-labeled gelatin (25 μg/mL; Millipore, ECM671) for 30 min and washed three times with PBS. To enhance the cell adhesion, cross-linked gelatin substrate was coated with collagen-I (50 μg/mL) for 30 min at 37 °C. Cells were seeded on the prepared substrate for 48 h and then fixed by freshly prepared 4% PFA. Degraded gelatin and F-actin (stained by CF680R-phalloidin) were visualized by an inverted spinning-disk confocal microscope. Percentages of degradation area relative to total cell area were analyzed using ImageJ software.

### Transwell assay

Transwell inserts with 8 μm porous membranes (Corning, 3421) were coated with Matrigel matrix (1 mg/mL in serum-free medium; Corning, 356237). Cells were seeded onto the upper chamber. Serum-containing medium as the chemoattractant was added to the lower chamber. After culturing for 24 h at 37 °C and 5% CO_2_, non-migrated cells were removed from the upper membrane, and migrated cells were stained with crystal violet. Migrated cells were visualized by an inverted microscope (Olympus IX71) and analyzed using ImageJ software.

### Statistical information

All datasets included at least three independent biological replicates. The results were presented as the mean ± standard error of the mean (SEM), and statistical analysis was performed using GraphPad Prism software. Statistical differences between two groups were analyzed using the Student's t-test. One-way analysis of variance (ANOVA) with Dunnett’s test was used to compare among three or more groups. The following significance levels were used: not significant (ns) for P > 0.1234, * for P < 0.0332, ** for P < 0.0021, *** for P < 0.0002, and **** for P < 0.0001. Statistical information, including exact P values, can be found in Table S1.

## Supplementary Information

Below is the link to the electronic supplementary material.

Supplementary file1 (DOCX 4967 KB)

## Data Availability

All data supporting the findings of this study are available within the paper and its Supplementary Information.
