# Supplementary material for: PI(3,4,5)P3-mediated Cdc42 activation regulates macrophage podosome assembly
Source: Cell Mol Life Sci. 2025 Mar 24;82(1):127. doi: 10.1007/s00018-025-05664-2 (PMC11933580; doi:10.1007/s00018-025-05664-2)
Supplement: Supplementary file 1 — Supplementary file1 (DOCX 4967 KB) [file 18_2025_5664_MOESM1_ESM.docx]

**Supplemental information**

**Figure S1.** (A-B) Western blot analysis to confirm WASP knockdown and rescue. (C) Degradation of Cy3-labeled gelatin in the indicated conditions. Representative images are stitched from 5x5 tile scan. Gelatin degradation activity of WASP-knockdown THP-1 macrophages is suppressed, compared to the control. Reintroductions of wildtype GFP-WASP, as opposed to GFP-WASP-3D restore the gelatin degradation (arrowheads). F-actin is stained by CF680R-phalloidin. (D) Representative images of transwell-migrated THP-1 macrophages in the indicated conditions. Crystal violet is used to stain the cells. All experiments have been independently repeated three times. Error estimates are S.E.M. The statistical information is in Table S1. Unpaired two-tailed Student’s t-test and one-way analysis of variance (ANOVA) are used for the statistical analysis. not significant, ns; P > 0.1234 and *P < 0.0332. Scale bars represent 50 μm in C and 200 μm in D.

**Figure S2.** (A) The RT-qPCR analysis of PIK3CA and PIK3CB in THP-1 macrophage. PIK3CB exhibits a higher expression level. (B) GFP-PIK3CB is enriched at the podosome. Podosome core and ring are identified by the staining of CF680R-phalloidin and anti-Paxillin, respectively. The insets represent the boxed regions. (C) Intensity line scans along the dashed line in B. (D-E) Western blot analysis of Cdc42-GTP level via GST-PBD pulldown. ML141 (20 µM) effectively blocks the Cdc42-GTP level. (F-G) Western blot analysis of Rac1-GTP level via GST-PBD pulldown. ML141 (20 µM) does not significantly alter the Rac1-GTP level. (H) Quantification of PIK3CB knockdown shown in Fig. 2I. (I-J) Dominant-negative Cdc42-T17N mutant, rather than wildtype Cdc42 blocks the podosome assembly in THP-1 macrophage. Densely polymerized F-actin puncta, a key feature of podosome core, are largely missing when Cdc42-T17N is expressed. (K-L) Western blot analysis of Rac1-GTP level via GST-PBD pulldown. EHT1864 (5 µM) effectively blocks the Rac1-GTP level. (M-N) EHT1864 treatment (5 µM) and DMSO control exhibit similar ratios of podosome-positive cells. The insets represent the boxed regions. (O-P) Western blot analysis of S473 phosphorylation of Akt. PIK3CB knockdown suppresses the level of pS473-Akt. All experiments have been independently repeated three times. Error estimates are S.E.M. The statistical information is in Table S1. Unpaired two-tailed Student’s t-test is used for the statistical analysis. not significant, ns; P > 0.1234; *P < 0.0332; ***P < 0.0002; and ****P < 0.0001. Scale bars represent 10 μm.

**Figure S3.** (A-B) GFP-PREX1 is not actively recruited to the plasma membrane upon the stimulation of EGF (100 ng/mL) in MEF. (C-D) EBFP-VAV3 is actively recruited to the plasma membrane upon the stimulation of EGF (100 ng/mL) in MEF. (E-F) GFP-PLEKHG2 is not actively recruited to the plasma membrane upon the stimulation of EGF (100 ng/mL) in MEF. (G-H) GFP-FGD3 is not actively recruited to the plasma membrane upon the stimulation of EGF (100 ng/mL) in MEF. (I-J) GFP-FGD2 is not actively recruited to the plasma membrane upon the stimulation of EGF (100 ng/mL) in MEF. (K-L) GFP-ARHGEF6 is not actively recruited to the plasma membrane upon the stimulation of EGF (100 ng/mL) in MEF. All experiments have been independently repeated three times. Error estimates are S.E.M. Scale bars represent 10 μm.

**Figure S4.** (A) The overall recruitment of EBFP-VAV1 to the cell-matrix interface is suppressed in PIK3CB-knockdown THP-1 macrophages, compared to the control. Podosome core and ring are identified by the staining of CF680R-phalloidin and anti-Paxillin, respectively. (B-C) EBFP-VAV1-W495L is not actively recruited to the plasma membrane upon the stimulation of EGF (100 ng/mL) in MEF. (D-E) Western blot analysis of pY160 VAV1 in the indicated conditions. The pY160 level remains diminished when VAV1-W495L is reintroduced to VAV1-knockdown THP-1 macrophages. All experiments have been independently repeated three times. Error estimates are S.E.M. The statistical information is in Table S1. One-way analysis of variance (ANOVA) is used for the statistical analysis. not significant, ns; P > 0.1234; *P < 0.0332; and **P < 0.0021. Scale bars represent 10 μm.

**Figure S5.** (A-K) Uncropped western blots.
